# Replacement soaking for human tankyrase 2 enables studies on substrate analogues and inhibitors

**DOI:** 10.1107/S2059798326006868

**Published:** 2026-07-29

**Authors:** Johan Pääkkönen, Sven T. Sowa, Chiara Bosetti, Lari Lehtiö

**Affiliations:** a Faculty of Biochemistry and Molecular Medicine and Biocenter Oulu University of Oulu Oulu Finland; University of Cambridge, United Kingdom

**Keywords:** replacement soaking, tankyrase, drug design, inhibitors, complex structure

## Abstract

Crystallography has facilitated the development of inhibitors in modern drug discovery. Complex structures are traditionally obtained by co-crystallization or soaking methods, but they are not always successful; co-crystallization may lead to selective crystallization of the apoprotein, and compounds cannot necessarily be soaked into preformed crystals. Successful endeavours in this field include human tankyrases TNKS1 and TNKS2, which are promising drug targets controlling a wide range of signalling events. In the case of TNKS2, apo crystals have a closed conformation in the donor loop, delineating the NAD^+^-binding active site, and compound soaking requires a large conformational change, which often damages the crystals and deteriorates the diffraction quality. This work describes a crystal form of TNKS2 that can be used efficiently in replacement soaking (also known as cross-soaking) when TNKS2 is co-crystallized with a low-affinity inhibitor. Replacement soaking is demonstrated with recently described nanomolar inhibitors as well as a nonhydrolysable analogue of NAD^+^, which all occupy the substrate-binding site. The obtained complex structures offer new insight into the binding modes of the inhibitors and the substrate NAD^+^, and comparison to previously published complex structures of closely related PARP proteins with NAD^+^ analogues reveals key characteristics of the NAD^+^-binding site of TNKS2. In cases where traditional methods fail to yield complex structures, the replacement soaking method provides an alternative method, which was demonstrated here to be necessary for elucidating the complex structures.

## Abstract 2

Replacement soaking, also known as cross-soaking, is a compelling method for solving complex structures in cases where traditional soaking and co-crystallization approaches fail. This method is demonstrated with human tankyrase 2, and new crystal structures of complexes with nanomolar inhibitors and an analogue of the substrate NAD^+^ are reported.

## 1. Introduction

Co-crystal structures provide critical data in the investigation of protein–ligand interactions that play a key role in, for instance, enzymology, protein engineering and drug development. Crystals of protein complexes are commonly created by co-crystallization or soaking methods (Hassell *et al.*, 2007 ▸; Müller, 2017 ▸; Wienen-Schmidt *et al.*, 2021 ▸). In co-crystallization, a protein–ligand complex is created, usually by simply mixing protein and ligand stocks, and crystallized. However, crystallizing the complex may require significantly different conditions to crystallizing only the apoprotein if the crystal packing is different, and thus new optimization of the crystallization conditions may be necessary. Furthermore, if the presence of organic solvent is required for the solubility of the ligand, it can affect the stability or crystallizability of the protein or disturb the crystallization process. Regardless of the binding affinity, co-crystallization attempts may also end up selectively crystallizing only the apoprotein. In soaking, the ligand of interest is introduced into existing crystals, typically of the apoprotein, but it is also possible to outcompete an existing ligand by replacement soaking, also known as cross-soaking (Ballone *et al.*, 2020 ▸; Hassell *et al.*, 2007 ▸; Pellegrini *et al.*, 2024 ▸; Singh *et al.*, 2013 ▸; Skarzynski & Thorpe, 2006 ▸). A common result of soaking attempts is that the crystals crack and dissolve when the strong binding of the ligand forces a conformational change that breaks the crystal lattice, or when organic solvent is introduced with the ligand. This can sometimes be mitigated by covalently cross-linking protein molecules in the crystal (Andersen *et al.*, 2009 ▸; Lusty, 1999 ▸; Parviainen *et al.*, 2025 ▸). Soaking can also fail if the ligand cannot enter the binding pocket because the pocket is not exposed to solvent channels or because the strong crystal packing prevents a required conformational change.

**Figure 1:**
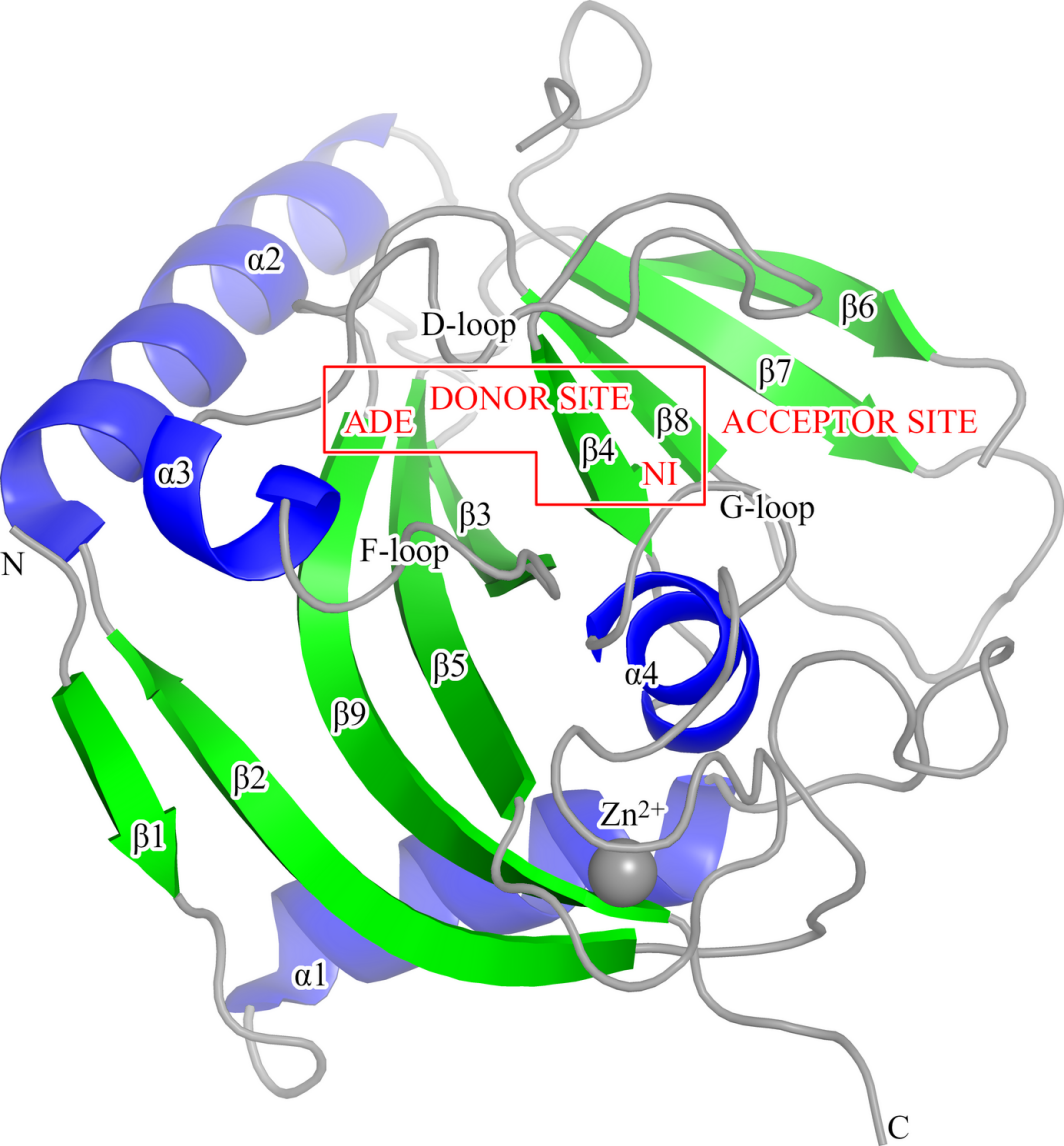
Tertiary structure of the TNKS2 catalytic domain in the apo form (PDB entry pdb_00003kr7; Karlberg *et al.*, 2010 ▸). The adenosine and nicotinamide subsites of the NAD^+^-binding donor site are labelled ADE and NI, respectively.

ADP-ribosylation is a post-translational modification that acts as a regulator of various biological processes (Lüscher *et al.*, 2022 ▸). It is formed by incorporating the ADP-ribose moiety of NAD^+^ into an acceptor protein, nucleic acid or antibiotic, and this reaction is catalysed by ADP-ribosyltransferases (ARTs). The most notable enzyme family in humans are the diphtheria toxin-like ARTs (ARTDs), which consist of 17 multidomain members that all share a conserved catalytic domain. Members of the family include poly-ADP-ribosyltransferases [formerly known as poly-(ADP-ribose) polymerases] PARP1 and PARP2 and tankyrases 1 and 2 (TNKS1 and TNKS2). These poly-ARTs attach ADP-ribose to target macromolecules, leading to the formation of mono-ADP-ribosyl (MAR) groups and subsequently elongate them to long poly-ADP-ribose (PAR) chains (Haikarainen *et al.*, 2014 ▸). In addition to the catalytic domain, the domain architecture of the tankyrases includes five target peptide-binding ankyrin repeat clusters (ARCs) and the sterile alpha motif (SAM) domain, via which the proteins assemble into a multimer and the catalytic domain is activated by subsequent dimerization (Jessop *et al.*, 2024 ▸; Pillay *et al.*, 2022 ▸; Sowa *et al.*, 2022 ▸). The general structure of the catalytic domain in ARTDs includes an NAD^+^-binding donor site and an acceptor site which binds the target protein and, in poly-ARTs, also MAR groups and PAR chains. In TNKS2, the catalytic domain includes a pair of antiparallel β-sheets that are surrounded by four α-helices and a structural Zn^2+^ ion in a loop region, and the donor site is defined by the β-sheets, α-helices α3 and α4, the donor loop (D-loop), the F-loop and the G-loop (Fig. 1 ▸; Haikarainen *et al.*, 2014 ▸).

**Figure 2:**
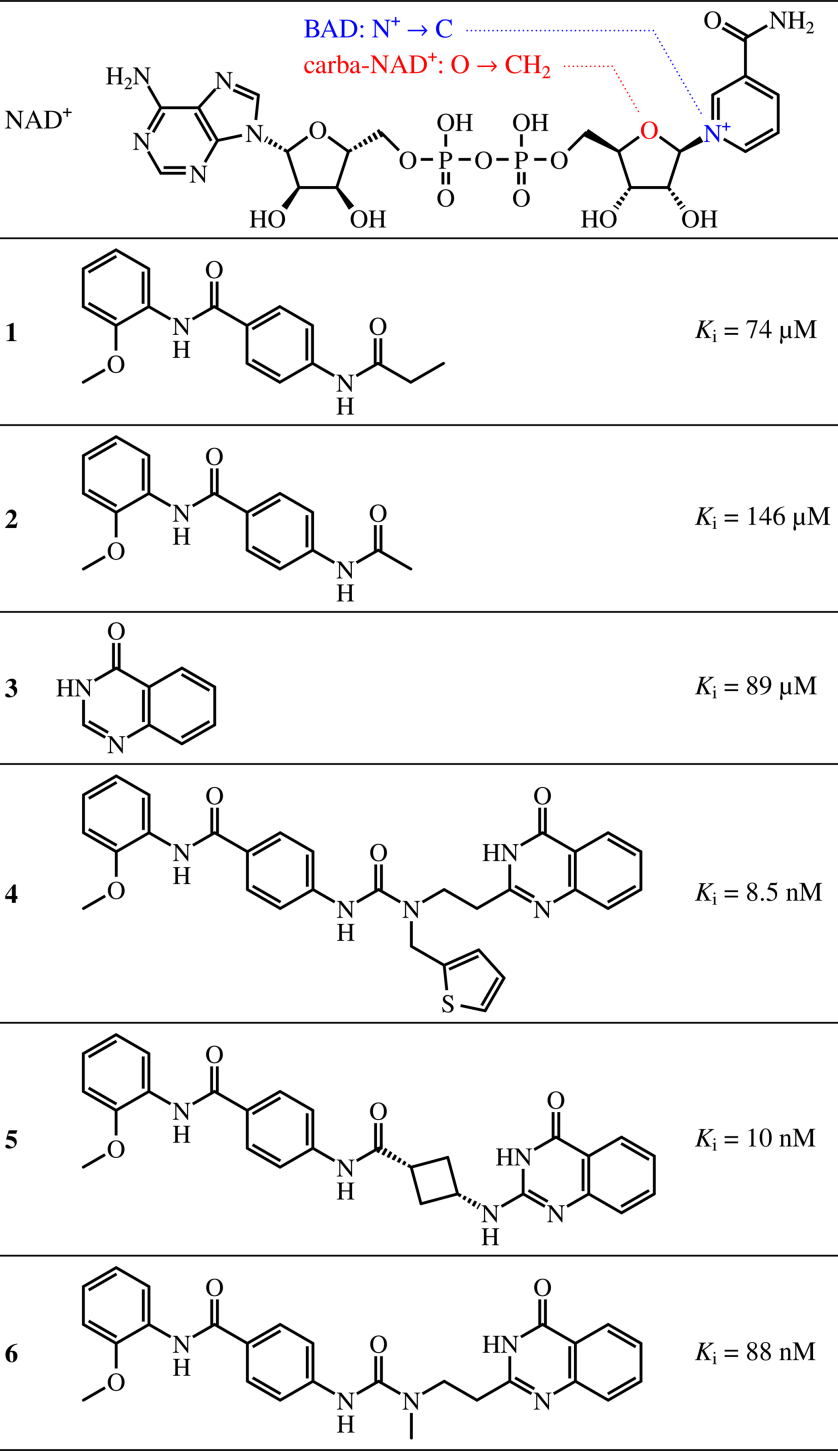
Structural formulae of the substrate NAD^+^ and compounds **1**–**6**. The nonhydrolysable analogues BAD (blue) and carba-NAD^+^ (red) are derived by substituting an atom of NAD^+^ as shown. Inhibition constants *K*_i_ were determined in our previous work (Sowa *et al.*, 2025 ▸).

Complex structures with the substrate NAD^+^ have not been possible to obtain for TNKS2 or any other ARTD. If co-crystallization or soaking is attempted with NAD^+^, the active enzyme will consume it before it can saturate the donor site and be observable by diffraction (Narwal *et al.*, 2012 ▸). A viable alternative is to use a nonhydrolysable analogue such as carbanicotinamide adenine dinucleotide (carba-NAD^+^) or benzamide adenine dinucleotide (BAD) (Fig. 2 ▸). Complex structures with these have been reported for the catalytic domains of other ARTDs, all of which are listed in Table 1 ▸. While the catalytic domain of TNKS2 (TNKS2^CAT^) is easy to crystallize in the apo form after limited proteolysis with chymotrypsin (Karlberg *et al.*, 2010 ▸; Narwal *et al.*, 2012 ▸; Ouzounthanasis *et al.*, 2026 ▸), soaking large compounds the size of NAD^+^ does not always work due to the large conformational change required for the D-loop lining the donor site.

Inhibitor research for ARTs traditionally focuses on targeting the NAD^+^-binding donor site. Well known PARP inhibitors include olaparib, niraparib, rucaparib and talazoparib, which target the nicotinamide subsite in PARP1/PARP2 and have been successful in cancer therapy (Roskoski, 2025 ▸; Wang *et al.*, 2025 ▸; Zeng *et al.*, 2024 ▸). There are currently no clinically available tankyrase-selective inhibitors, although some have proceeded to clinical trials (Chen *et al.*, 2024 ▸; Lieu *et al.*, 2025 ▸), and thus their discovery and development is important. In this context, solving crystal structures of the tankyrase catalytic domain in complex with inhibitors has been a valuable strategy. In our previous study (Sowa *et al.*, 2025 ▸), we optimized previously known dual-site binders (Bregman *et al.*, 2013 ▸; Nathubhai *et al.*, 2017 ▸) by designing new linkers between the adenosine-mimicking (compounds **1** and **2**; structural formulae in Fig. 2 ▸) and nicotinamide-mimicking (compound **3**) moieties and maximizing the affinity for TNKS2^CAT^, which resulted in several nanomolar inhibitors, including compounds **4**–**6**. Although complex structures of TNKS2^CAT^+**2**+**3** and TNKS2^CAT^+**4** were solved, multiple other complex structures could not be obtained. We discovered that compounds **1** and **2** bind with low affinity, and thus they should be possible to outcompete in the protein crystal with the newly described strong inhibitors. As a result, in this study we report a successful method of replacement soaking of the nanomolar inhibitors **4**–**6** spanning both the nicotinamide- and adenosine-binding subsites as well as the nonhydrolysable NAD^+^ analogue BAD into TNKS2^CAT^, the latter of which is currently the best representative structure of substrate-bound tankyrase.

## 2. Materials and methods

### 2.1. Cloning and protein production

Gene cloning and protein production are described in previous work (Sowa & Lehtiö, 2022 ▸; Sowa *et al.*, 2025 ▸). The final TNKS2^CAT^ batch was in a buffer consisting of 20 m*M* HEPES pH 6.8, 350 m*M* NaCl, 5%(*v*/*v*) glycerol, 0.5 m*M* TCEP. Chymotrypsin was added in a 1:100 weight ratio to TNKS2^CAT^, and the solution was incubated for an hour at room temperature and then concentrated to a TNKS2^CAT^ concentration of 7.7 mg ml^−1^. A 5.5 mg ml^−1^ dilution was prepared by diluting with a similar buffer such that the final HEPES concentration was 30 m*M* and the pH was 7.0, with the other buffer components being the same.

### 2.2. Protein crystallization

The TNKS2^CAT^ co-crystallization described by Sowa *et al.* (2025 ▸) was repeated (Table 2 ▸). A crystallization plate was prepared with reservoirs consisting of 20–26%(*w*/*v*) PEG 3350, 200 m*M* Li_2_SO_4_, 100 m*M* Tris pH 8.5. Crystallization droplets were prepared by manually pipetting protein solution (7.7 or 5.5 mg ml^−1^) and reservoir solution supplemented with 1 m*M* compound **1** or **2** and 1%(*v*/*v*) DMSO in three protein:reservoir drop ratios (1:2, 1:1 and 2:1) with a final droplet volume of 1 µl. In other words, compound **1** or **2** was added only to the droplets with the reservoir solution and not used in the reservoirs. Small needle-like crystals grew consistently in all conditions with either compound, although compound **1**co-crystals were larger and were thus used in replacement soaking experiments. Based on the results in the earlier work, they were expected to diffract to around 2.5 Å resolution.

### 2.3. Replacement soaking

The complexes with compounds **4**–**6** and BAD were created by placing compound **1** co-crystals in a 2 µl droplet of soaking solution containing 100 µ*M* of either compound **4**, **5** or **6** or 10 m*M* BAD. Full compositions of the soaking solutions are shown in Table 2 ▸. A rather large droplet volume was used so that the concentrations of protein and compound **1** would be very low and compound **1** would be efficiently replaced. Concentrations of compounds **4**–**6** were limited by their low solubility, stock concentrations being 10 m*M* in DMSO, and no more than 1%(*v*/*v*) DMSO was used because it would destabilize the protein. After soaking overnight (at least 16 h) at 4°C over the original reservoir, the crystals were transferred to cryoprotective solution (Table 2 ▸), immediately mounted in SPINE pins and flash-cooled in liquid nitrogen. The temperature was the same as that at which the crystals were grown, and in general keeping the temperature constant is the safest option for the crystals. The low temperature also decreases the rate of ligand replacement, requiring a longer soaking time than at room temperature. Without knowing the binding kinetics, a long soaking time, of the order of several hours or even days, is preferred in any case to maximize ligand occupancy (Müller, 2017 ▸; Wienen-Schmidt *et al.*, 2021 ▸).

### 2.4. X-ray crystallography

X-ray diffraction data (Lehtiö & Pääkkönen, 2024 ▸) were collected at European synchrotrons (Table 3 ▸). Datasets for the compound **4**, **5** and **6** complex structures were collected on the ID30A-1 (MASSIF-1) beamline (Bowler *et al.*, 2015 ▸) at the European Synchrotron Radiation Facility (ESRF), and datasets for the BAD complex structure were collected on the I04 beamline at Diamond Light Source (DLS). Preliminary data were also collected on the ID23-1 beamline (Nurizzo *et al.*, 2006 ▸) at the ESRF. As with PDB entry pdb_00007ojo in the previous work (Sowa *et al.*, 2025 ▸), multiple isomorphous datasets from identical individual crystals were merged, which maximized the redundancy and led to higher signal to noise in the highest resolution bins.

### 2.5. Structure determination

Raw data were processed with the *XDS* program package (Kabsch, 2010 ▸) and merged with *XSCALE*. Three datasets were merged for compounds **4** and **5** and BAD, and five for compound **6**. The final unit-cell dimensions were averages of the refined dimensions for the individual datasets. The data were phased using molecular replacement in *Phaser* (McCoy *et al.*, 2007 ▸) run via the *CCP*4*i*2 interface (Agirre *et al.*, 2023 ▸, Potterton *et al.*, 2018 ▸) with the complex structure PDB entry pdb_00007ojo (Sowa *et al.*, 2025 ▸) as the search model, containing two protein monomers in the asymmetric unit. Structure refinement was performed with *REFMAC*5 (Kovalevskiy *et al.*, 2018 ▸; Murshudov *et al.*, 2011 ▸). Riding hydrogens were used in refinement but were omitted from the result files, individual isotropic *B* factors were refined for all non-H atoms and local noncrystallographic symmetry (NCS) restraints were used to stabilize the geometry of the protein. As the electron density of BAD appeared to be weak, the occupancies of BAD molecules were refined as incomplete occupancy groups. Geometry restraints for compounds **4**–**6** and BAD were calculated with *PRODRG* (Schüttelkopf & van Aalten, 2004 ▸). The structures were edited manually in *Coot* (Emsley *et al.*, 2010 ▸) and visualized in *PyMOL* (version 2.5.0; Schrödinger). Water molecules were added using the ‘Find Waters’ tool in *Coot*, checked manually and deleted if the chemical environment was inappropriate or if the electron density was unconvincing. Sulfate ions were added manually in large positive difference-map peaks where the chemical environment was appropriate for them. Statistics of structure refinement are shown in Table 4 ▸.

## 3. Results

### 3.1. Background

In the previous study (Sowa *et al.*, 2025 ▸), we pretreated TNKS2^CAT^ with chymotrypsin and co-crystallized it with the adenosine-mimicking compounds **1** and **2**, and then soaked the nicotinamide-mimicking compound **3** into compound **2**co-crystals. In the resulting complex structure (PDB entry pdb_00007ojo) both compounds were bound to their respective subsites, and the D-loop was in the open conformation. Our attempts to co-crystallize TNKS2^CAT^ with BAD and higher affinity inhibitors related to **1** and **2** either failed to produce macrocrystals or resulted in a crystallized apo form with a closed D-loop in a different crystal form (data not shown), suggesting that slow nucleation and growth of co-crystals with adenosine site binders require a weakly binding ligand and only partial ligand occupancy at the nucleation stage. However, we were able to soak compound **4** into apo TNKS2^CAT^ crystals and solve the complex structure (pdb_00008b6m). Later, considering that **1** and **2** are low-affinity binders in contrast to previously co-crystallized adenosine-site binders with nanomolar affinity (Anumala *et al.*, 2017 ▸; Haikarainen *et al.*, 2016 ▸; Leenders *et al.*, 2021 ▸; Qiu *et al.*, 2014 ▸; Sowa & Lehtiö, 2022 ▸; Voronkov *et al.*, 2013 ▸; Waaler *et al.*, 2020 ▸), we presumed that obtaining the complex structures with compounds **4**–**6** should be possible by replacement soaking after co-crystallization. Similarly, soaking with BAD might yield a complex structure with an analogue of the elusive NAD^+^.

**Figure 3:**
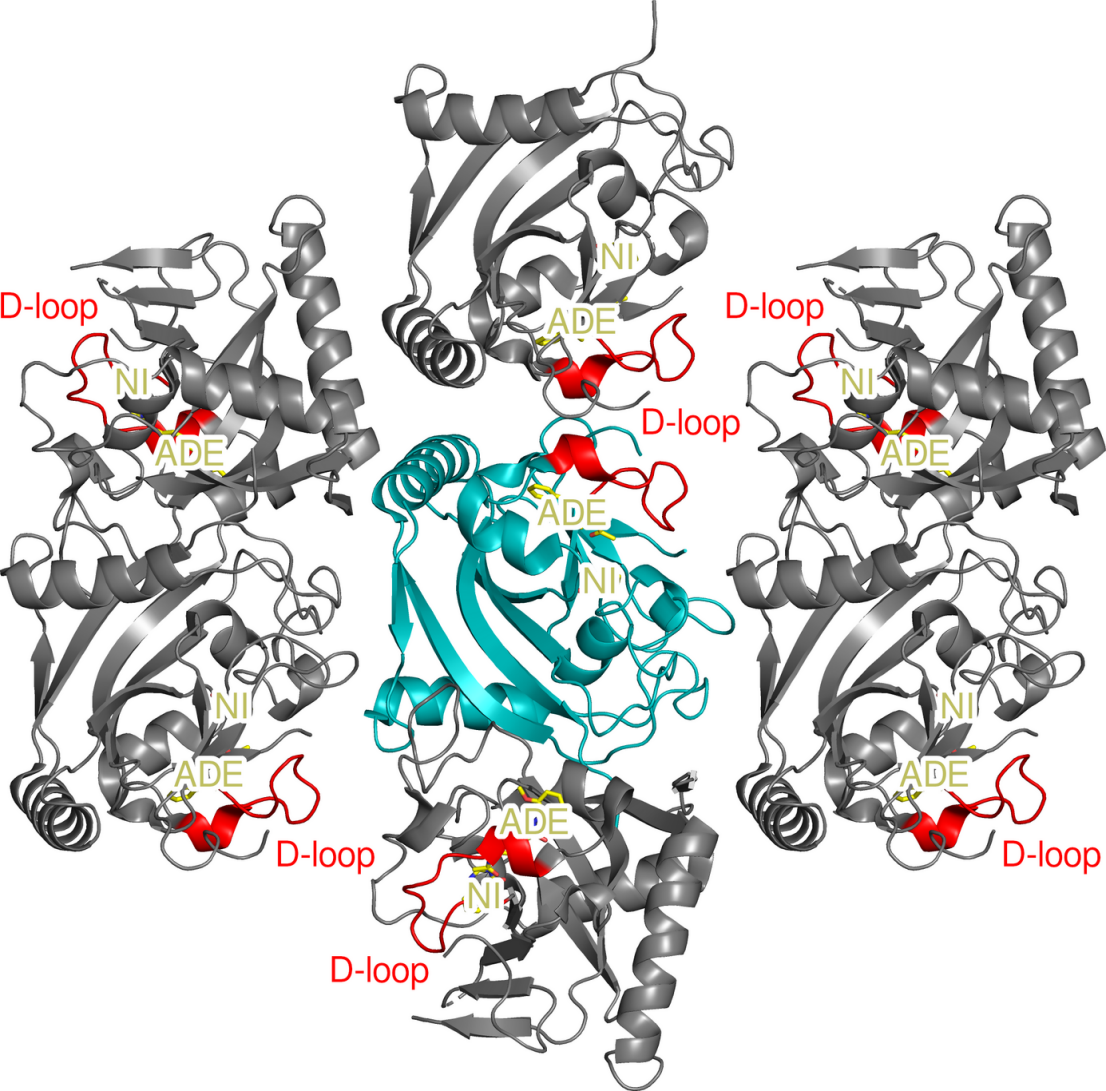
Crystal contacts of a TNKS2^CAT^ monomer in the considered crystal form. The crystal structure is PDB entry pdb_00007ojo (Sowa *et al.*, 2025 ▸). A protein monomer (cyan) is surrounded by seven neighbouring molecules (six roughly in the same plane shown in gray, the seventh one behind not shown for clarity). The D-loop (highlighted in red) is packed against the loop region preceding the β7 strand of a neighbouring molecule, which likely plays a major role in the stabilization of the crystal lattice. The adenosine and nicotinamide subsites of the donor site are labelled ADE and NI, respectively.

TNKS2^CAT^ has been crystallized in various crystal forms by multiple research groups. A search of the Protein Data Bank (PDB; Berman *et al.*, 2000 ▸) yields crystal structures in five different space groups, of which *C*222_1_ and *P*2_1_2_1_2_1_ are the two most common. Furthermore, there are two distinct *P*2_1_2_1_2_1_ crystal forms which feature different crystal contacts, unit cells (volumes 470 000 Å^3^ versus 900 000 Å^3^) and solvent contents (49% versus 46%). The crystals considered in this study as well as PDB entry pdb_00007ojo belong to the former group where the somewhat looser crystal packing (shown in Fig. 3 ▸) presumably allows more flexibility in the D-loop while crystal contacts stabilize the open D-loop conformation, and therefore the original co-crystallized ligand is easier to replace.

### 3.2. Crystallization and ligand-replacement strategies

The replacement soaking experiments were successful, and we solved four new complex structures of TNKS2^CAT^. Compared with the previous TNKS2^CAT^+**2**+**3** crystal structure (PDB entry pdb_00007ojo, 2.30 Å resolution), crystals of two complexes diffracted to similar resolution (2.30 and 2.35 Å), while the diffraction of the other two was significantly weaker (2.80 and 2.90 Å). It is expected that the soaking process forces conformational changes that cause disturbances in the lattice and reduce the diffracting power, although a significant drop in resolution is undesired in most applications. Regardless, in this case we were able to determine the structures and place the compounds in the donor sites with high confidence.

We attempted to perform replacement soaking with BAD in three subsequent steps (soaking times of 24, 72 and 24 h). The crystals appeared to remain intact, but the manipulation steps damaged the crystals and reduced the data resolution to 3.2 Å in the best case (data not shown). We also attempted to create the structure of the apo form with the open D-loop conformation by washing the co-crystals in a solution containing no BAD or inhibitors. One washing step overnight was not sufficient to remove the original inhibitor, and three washing steps as above severely reduced the data resolution to about 3.7 Å, but the pocket then appeared to be empty in the low-resolution maps (data not shown). We did not try shorter soaking times as the soaking process was expected to be slow at 4°C.

### 3.3. Complex structures with inhibitors

**Figure 4:**
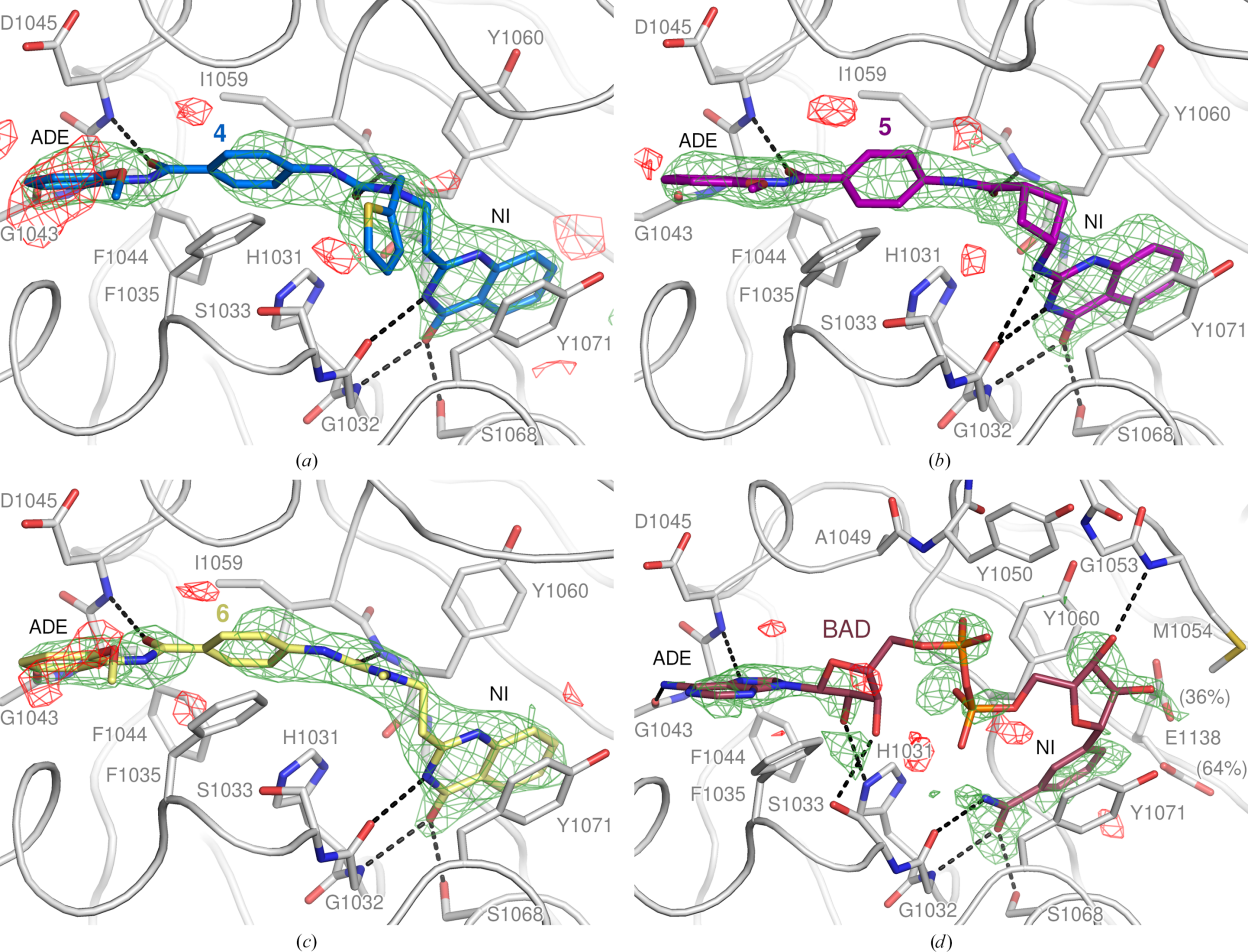
Omit maps of compounds (*a*) **4**, (*b*) **5**, (*c*) **6** and (*d*) BAD in the NAD^+^-binding donor site of TNKS2^CAT^. The adenosine and nicotinamide subsites are labelled ADE and NI, respectively. Hydrogen bonds up to 3.2 Å in length are shown as dashed lines. The *mF*_o_ − *DF*_c_ omit maps contoured at ±3σ are shown around the ligands in green (positive) and red (negative). Complete 2*mF*_o_ − *DF*_c_ and *mF*_o_ − *DF*_c_ maps of the ligand environments are shown in Supplementary Fig. S1. In (*d*), the two alternative conformations of the catalytic Glu1138 are shown with their mean occupancies in the two chains.

Complex structures with compounds **4**, **5** and **6** have very clear electron densities for the compounds with little uncertainty (Figs. 4 ▸*a*–4 ▸*c*, Supplementary Figs. S1*a*–S1*c*). The binding modes are as expected based on the original inhibitor design, and adenosine- and nicotinamide-mimicking moieties reflect the binding modes observed for fragment compounds **2** and **3** in PDB entry pdb_00007ojo. The placement of the compound in TNKS2^CAT^+**4** also matches PDB entry pdb_00008b6m, with small differences in the orientations of the adenine-mimicking *ortho*-methoxyphenyl group and the thiophene group. As in PDB entry pdb_00008b6m, the expected hydrogen bond between the carbonyl O atom of the urea-based linker and the backbone NH of Tyr1060 is rather long (3.40 Å on average) and weak. As replacing the optimally bound water molecule that occupies this region in the apo structure (PDB entry pdb_00003kr7; Karlberg *et al.*, 2010 ▸) with another hydrogen-bond acceptor at a suboptimal distance is unfavourable (Chen *et al.*, 2016 ▸), the long hydrogen bond is expected to negatively affect the affinity. In comparison, the corresponding distances in TNKS2^CAT^+**5** and TNKS2^CAT^+**6** are 3.25 and 3.22 Å, respectively, which are still longer than the optimal distance of approximately 3.0 Å (calculated using the data by Taylor *et al.*, 1984 ▸), but the compounds are too rigid to allow the carbonyl to move closer. Conversely, the thiophene group of compound **4** pulls the urea-based linker back slightly and extends to fill a small hydrophobic pocket formed by Pro1034, Phe1035, Tyr1071 and Ile1075, which reduces unfavourable interactions between hydrophobic groups and water, and may thus be the major contributing factor to the high affinity. Despite the lower data resolution (2.8 versus 1.6 Å), the electron density of the compound in TNKS2^CAT^+**4** is better than in the previously reported structure (PDB entry pdb_00008b6m). Otherwise, in the previous structure the electron density of the protein is clearer, but in this case the protein was also crystallized in a different *C*222_1_ crystal form which in general diffracts more strongly. As described in Section 3.5, the D-loop conformations in this structure and the previous TNKS2^CAT^+**4** structure are significantly different.

### 3.4. Substrate NAD^+^ analogue complex structure with TNKS2

**Figure 5:**
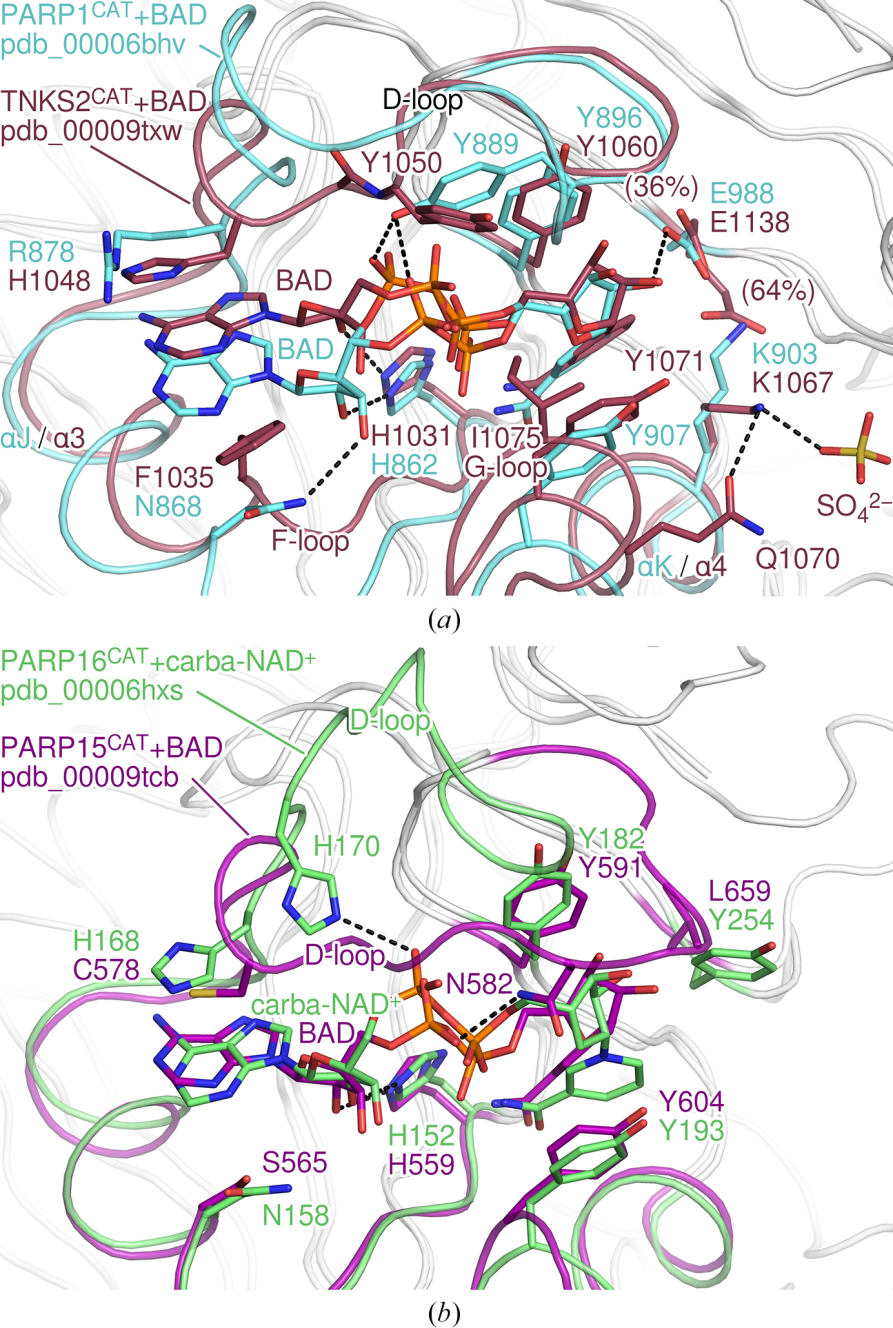
Comparison of the donor sites in human ARTD complex structures containing NAD^+^ analogues. (*a*) Poly-ARTDs PARP1 and TNKS2. Notable structural differences include the conformations of the D-loop, the narrower adenosine subsite restricted by the F-loop in TNKS2, and the longer G-loop in TNKS2. (*b*) Mono-ARTDs PARP15 and PARP16. The donor sites are shaped similarly to each other as well as to PARP1, except for the different conformations of the D-loop.

In comparison to the solved inhibitor complexes, the substrate analogue BAD had weaker electron density in the TNKS2^CAT^+BAD complex structure (Fig. 4 ▸*d*, Supplementary Fig. S1*d*), but as the binding mode was known based on the similar PARP1 (PDB entry pdb_00006bhv), BAD could be placed confidently. The refined occupancies of the BAD molecules in the two chains were 80% and 87%. Comparing the structure with the previous complex structures of human ARTDs listed in Table 1 ▸, the shape of the donor site is characteristically different in TNKS2, whereas the donor sites of PARP1, PARP15 and PARP16 are relatively similar to one another, with differences in the conformations of the D-loop and in unconserved amino-acid residues (Fig. 5 ▸). Notably, the glutamate of the catalytic His–Tyr–Glu triad in the poly-ARTDs PARP1 and TNKS2 is a leucine or tyrosine in the mono-ARTDs PARP15 and PARP16, respectively. In TNKS2^CAT^+BAD the glutamate (Glu1138) exists in two alternative conformations: the conformation pointing to the ribose of BAD as in PARP1 is the minor form with 36% mean occupancy, while the major conformation with 64% mean occupancy points away towards Lys1067 (Figs. 4 ▸*d* and 5 ▸*a*). In PARP1, the 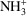 group of the corresponding lysine occupies the space of this major conformation, whereas in TNKS2^CAT^+BAD Lys1067 points towards Gln1070 and a sulfate ion (Fig. 5 ▸*a*). As the sulfate has been introduced in the crystallization solution at a high concentration, it is absent under physiological conditions, and therefore this double conformation of Glu1138 may be merely a crystallization artefact.

The D-loop conformations and lengths are notably different in the human ARTDs. Compared with PARP1, in PARP15 (PDB entry pdb_00009tcb), TNKS2^CAT^+BAD and PARP16 (PDB entry pdb_00006hxs) the D-loop is two, four and three residues shorter, respectively. In PARP15 the D-loop is placed relatively close to BAD, forming an additional hydrogen bond to it via an asparagine, whereas in PARP16 it turns away from the donor site, forming only a single hydrogen bond to carba-NAD^+^ via a histidine (Fig. 5 ▸*b*). In TNKS2^CAT^+BAD, the D-loop is placed next to the phosphates of BAD and does not extend as far above the nicotinamide subsite as in PARP15 (Fig. 5 ▸*a*). Instead of a tyrosine in PARP1 forming a short hydrogen bond to one of the phosphates, in TNKS2 a weak hydrogen bond is formed by the backbone NH of Tyr1050.

A detailed comparison of PARP1 and TNKS2 reveals other subtle differences in the donor site (Fig. 5 ▸*a*). The beginning of the α3 helix in TNKS2 (αJ in PARP1) is a shorter F-loop, and in consequence Phe1035 points towards the adenine pocket, narrowing it, and the adenine of BAD appears to be pushed up towards the D-loop, where the adenine is stacked against His1048 instead of arginine in PARP1. The loop region after the α4 helix in TNKS2 (αK in PARP1) is a longer G-loop, which introduces Gly1074, Ile1075 and Gly1076 delineating the pocket next to the phosphates of BAD. The preceding Tyr1071 is also positioned higher up towards the nicotinamide, making the nicotinamide subsite on average 0.8 Å narrower than in the other ARTDs.

### 3.5. Comparison of D-loop conformations in TNKS2 complex structures

**Figure 6:**
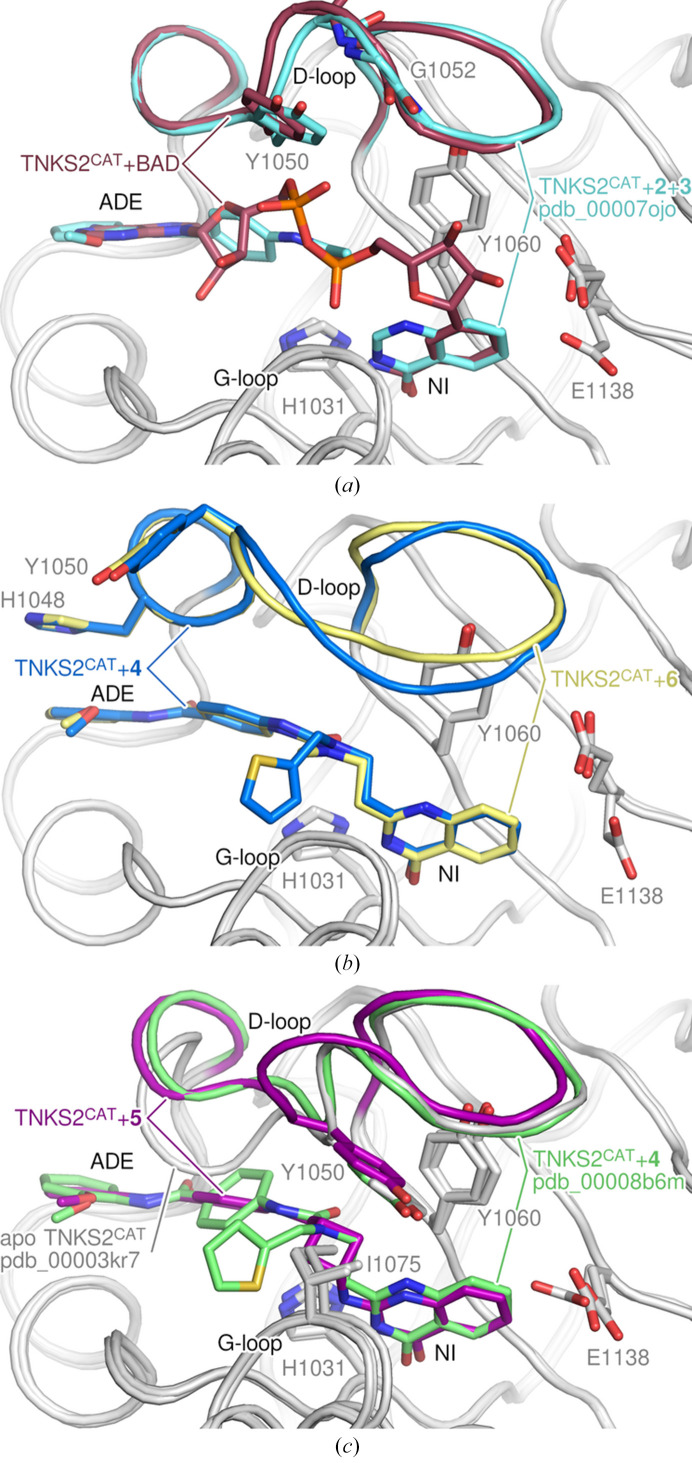
The D-loop (coloured range Glu1046–Gly1058 at the top of each panel) is observed in three distinct conformations in the TNKS2^CAT^ complex structures. The adenosine and nicotinamide subsites of the NAD^+^-binding donor site are labelled ADE and NI, respectively. (*a*) In TNKS2^CAT^+BAD the D-loop is completely open as in PDB entry pdb_00007ojo (TNKS2^CAT^+**2**+**3**). (*b*) In TNKS2^CAT^+**4** and TNKS2^CAT^+**6** the D-loop covers the nicotinamide subsite in a way that would clash with BAD. (*c*) In TNKS2^CAT^+**5** the D-loop is partially closed as in PDB entry pdb_00008b6m (TNKS2^CAT^+**4**), and this conformation most resembles the closed conformation in the apo form (PDB entry pdb_00003kr7).

The conformations of the flexible D-loop are different in the TNKS2^CAT^ crystal structures (Fig. 6 ▸). In contrast to the apo form (PDB entry pdb_00003kr7), where the D-loop completely closes the NAD^+^-binding site, in all structures presented in this work it has opened to accommodate the large ligand, and the flexibility is shown by weak electron density and large atomic *B* factors from Tyr1050 to Gly1056. The D-loop conformations are best characterized by the orientation of the flexible side chain of Tyr1050. In the TNKS2^CAT^+BAD structure the D-loop is completely open as in PDB entry pdb_00007ojo (TNKS2^CAT^+**2**+**3**), and Tyr1050 points into the bulk solvent in the general direction of Gly1052 (Fig. 6 ▸*a*). In TNKS2^CAT^+**4** and TNKS2^CAT^+**6** the D-loop has shifted to cover the nicotinamide subsite, and Tyr1050 has turned away to the same direction as His1048 (Fig. 6 ▸*b*). Here the deviations of C^α^ positions in alignment to the apo form are the largest: up to 6.1 Å at Tyr1050. Also, the electron density of the D-loop is the weakest as the loop conformation has changed the most in soaking, indicating that significant flexibility is introduced by the replacement soaking process. Presumably due to the dynamic conformations of the D-loop and related disturbance of the crystal lattice, these crystals also had the weakest diffraction of all. Finally, in TNKS2^CAT^+**5** the D-loop is partially closed as in PDB entry pdb_00008b6m (TNKS2^CAT^+**4**) in a way that resembles the apo form, and the nicotinamide subsite is obscured by Tyr1050, which is packed against the ligand via nonpolar interactions and points towards Ile1075 in the G-loop (Fig. 6 ▸*c*).

## 4. Conclusions

As demonstrated, replacement soaking is a successful method for solving new complex structures. We determined new TNKS2^CAT^ complex structures with BAD and compounds **4**–**6**, and while that of TNKS2^CAT^+**4** was already known and published (PDB entry pdb_00008b6m; Sowa *et al.*, 2025 ▸), the new structure had better electron density for the compound. Compared with the crystal structure with no replacement soaking performed (PDB entry pdb_00007ojo; Sowa *et al.*, 2025 ▸), in some cases there was an unfortunate decrease in data resolution. As with any crystal manipulation, it is inevitable that the data quality suffers, but the extent of quality loss and consistency is highly dependent on the protein itself and the crystal system. Regardless, if the desired complex structures turn out to be unobtainable using conventional co-crystallization or soaking techniques, but co-crystallization with another lower affinity substrate analogue or inhibitor is viable, replacement soaking is a compelling approach with great potential to produce useful results.

The complex structure TNKS2^CAT^+BAD reported in this work is currently the most accurate model of how TNKS2 binds the substrate NAD^+^. Comparison to other human ARTDs in complex with NAD^+^ analogues shows how the NAD^+^-binding site is unique in TNKS2, which will be useful information in future inhibitor development. The complex structures with dual-site inhibitors **4**, **5** and **6** confirm that the assumptions made about the binding modes in their design were correct and show an interesting aspect of D-loop dynamics. The flexibility of the D-loop could also be a major contributing factor to the affinity and binding kinetics of different inhibitors, which should be considered in future drug development, although based on the limited data there is no clear correlation between the observed D-loop conformations and the measured affinities.

## Supplementary Material

PDB reference: catalytic domain of human tankyrase 2, complex with compound **4**, pdb_00009txt

PDB reference: complex with compound **5**, pdb_00009txu

PDB reference: complex with compound **6**, pdb_00009txv

PDB reference: complex with benzamide adenine dinucleotide, pdb_00009txw

Supplementary Figure S1. DOI: 10.1107/S2059798326006868/rr5265sup1.pdf

Raw diffraction images.: https://doi.org/10.23729/ee61d6dd-5100-4fee-bb21-bf78050d240c

Data-collection session at the ESRF.: https://doi.org/10.15151/ESRF-ES-1309325308

## Figures and Tables

**Table 1:** Previously published complex structures of catalytic domains of ARTDs with an NAD^+^ analogue in a binding site

PDB code	ARTD	NAD^+^ analogue in donor site	NAD^+^ analogue in acceptor site	Reference
pdb_00001a26	Chicken PARP1	—	Carba-NAD^+^ (partial)	Ruf *et al.* (1998 ▸)
pdb_00006bhv	Human PARP1	BAD	—	Langelier *et al.* (2018 ▸)
pdb_00006hxs	Human PARP16	Carba-NAD^+^	—	T. Karlberg, A. F. Pinto, A. G. Thorsell & H. Schüler (unpublished work)
pdb_00009bpy	Human PARP1	BAD	Carba-NAD^+^	Langelier *et al.* (2024 ▸)
pdb_00009dmc	Human PARP1	BAD	ADP-ribose	Langelier *et al.* (2024 ▸)
pdb_00009tcb	Human PARP15	BAD	—	Tuovinen *et al.* (2025 ▸)

**Table 2:** Crystallization

Crystal structure	TNKS2^CAT^+**4**	TNKS2^CAT^+**5**	TNKS2^CAT^+**6**	TNKS2^CAT^+BAD
Method	Vapour diffusion, sitting drop	Vapour diffusion, sitting drop	Vapour diffusion, sitting drop	Vapour diffusion, sitting drop
Plate type	Swissci 3 Lens	Swissci 3 Lens	Swissci 3 Lens	Swissci 3 Lens
Temperature (°C)	4	4	4	4
Protein concentration (mg ml^−1^)	5.5	5.5	5.5	5.5
Buffer composition of protein solution	30 m*M* HEPES pH 7.0, 350 m*M* NaCl, 5%(*v*/*v*) glycerol, 0.5 m*M* TCEP	30 m*M* HEPES pH 7.0, 350 m*M* NaCl, 5%(*v*/*v*) glycerol, 0.5 m*M* TCEP	30 m*M* HEPES pH 7.0, 350 m*M* NaCl, 5%(*v*/*v*) glycerol, 0.5 m*M* TCEP	30 m*M* HEPES pH 7.0, 350 m*M* NaCl, 5%(*v*/*v*) glycerol, 0.5 m*M* TCEP
Composition of reservoir solution	20%(*w*/*v*) PEG 3350, 200 m*M* Li_2_SO_4_, 100 m*M* Tris pH 8.5	20%(*w*/*v*) PEG 3350, 200 m*M* Li_2_SO_4_, 100 m*M* Tris pH 8.5	20%(*w*/*v*) PEG 3350, 200 m*M* Li_2_SO_4_, 100 m*M* Tris pH 8.5	22%(*w*/*v*) PEG 3350, 200 m*M* Li_2_SO_4_, 100 m*M* Tris pH 8.5
Reservoir solution in drop	20%(*w*/*v*) PEG 3350, 200 m*M* Li_2_SO_4_, 100 m*M* Tris pH 8.5, 1%(*v*/*v*) DMSO, 1 m*M* compound **1**	20%(*w*/*v*) PEG 3350, 200 m*M* Li_2_SO_4_, 100 m*M* Tris pH 8.5, 1%(*v*/*v*) DMSO, 1 m*M* compound **1**	20%(*w*/*v*) PEG 3350, 200 m*M* Li_2_SO_4_, 100 m*M* Tris pH 8.5, 1%(*v*/*v*) DMSO, 1 m*M* compound **1**	22%(*w*/*v*) PEG 3350, 200 m*M* Li_2_SO_4_, 100 m*M* Tris pH 8.5, 1%(*v*/*v*) DMSO, 1 m*M* compound **1**
Volume and ratio of drop (protein:reservoir)	1 µl, 1:1	1 µl, 1:1	1 µl, 1:1	1 µl, 2:1
Volume of reservoir (µl)	50	50	50	50
Composition of the soaking solution	20%(*w*/*v*) PEG 3350, 200 m*M* Li_2_SO_4_, 100 m*M* Tris pH 8.5, 5%(*v*/*v*) glycerol, 1%(*v*/*v*) DMSO, 100 µ*M* compound **4**	20%(*w*/*v*) PEG 3350, 200 m*M* Li_2_SO_4_, 100 m*M* Tris pH 8.5, 5%(*v*/*v*) glycerol, 1%(*v*/*v*) DMSO, 100 µ*M* compound **5**	20%(*w*/*v*) PEG 3350, 200 m*M* Li_2_SO_4_, 100 m*M* Tris pH 8.5, 5%(*v*/*v*) glycerol, 1%(*v*/*v*) DMSO, 100 µ*M* compound **6**	20%(*w*/*v*) PEG 3350, 200 m*M* Li_2_SO_4_, 100 m*M* Tris pH 8.5, 5%(*v*/*v*) glycerol, 1%(*v*/*v*) DMSO, 10 m*M* BAD
Composition of the cryoprotectant	20%(*w*/*v*) PEG 3350, 200 m*M* Li_2_SO_4_, 100 m*M* Tris pH 8.5, 20%(*v*/*v*) glycerol, 1%(*v*/*v*) DMSO, 100 µ*M* compound **4**	20%(*w*/*v*) PEG 3350, 200 m*M* Li_2_SO_4_, 100 m*M* Tris pH 8.5, 20%(*v*/*v*) glycerol, 1%(*v*/*v*) DMSO, 100 µ*M* compound **5**	20%(*w*/*v*) PEG 3350, 200 m*M* Li_2_SO_4_, 100 m*M* Tris pH 8.5, 20%(*v*/*v*) glycerol, 1%(*v*/*v*) DMSO, 100 µ*M* compound **6**	20%(*w*/*v*) PEG 3350, 200 m*M* Li_2_SO_4_, 100 m*M* Tris pH 8.5, 20%(*v*/*v*) glycerol, 1%(*v*/*v*) DMSO
Drop setting	Manual	Manual	Manual	Manual
Seeding	No	No	No	No

**Table 3:** Data collection and processing Values in parentheses are for the highest resolution shell.

	TNKS2^CAT^+**4**	TNKS2^CAT^+**5**	TNKS2^CAT^+**6**	TNKS2^CAT^+BAD
Diffraction source	ID30A-1, ESRF	ID30A-1, ESRF	ID30A-1, ESRF	I04, DLS
Wavelength (Å)	0.96546	0.96546	0.96546	0.95374
Temperature (K)	100	100	100	100
Detector	PILATUS3 6M	PILATUS3 6M	PILATUS3 6M	EIGER2 XE 16M
Crystals used†	3	3	5	3
Crystal-to-detector distance† (mm)	677.16/827.75/622.75	589.90/543.61/456.43	827.75/827.75/635.84/531.31/827.75	292.03/359.97/365.00
Total rotation range† (°)	180/180/180	180/180/180	180/180/360/360/180	360/360/360
Rotation per image† (°)	0.2/0.2/0.2	0.2/0.2/0.2	0.2/0.2/0.2/0.2/0.2	0.1/0.1/0.1
Exposure time per image† (s)	0.0264/0.0333/0.0240	0.0225/0.0200/0.0200	0.0338/0.0340/0.0200/0.0200/0.0333	0.0027/0.0039/0.0040
Space group	*P*2_1_2_1_2_1_	*P*2_1_2_1_2_1_	*P*2_1_2_1_2_1_	*P*2_1_2_1_2_1_
*a*, *b*, *c* (Å)	41.71, 76.30, 148.98	41.68, 76.50, 149.02	41.79, 76.43, 149.06	41.86, 76.61, 148.91
α, β, γ (°)	90, 90, 90	90, 90, 90	90, 90, 90	90, 90, 90
Mosaicity† (°)	0.107/0.172/0.090	0.072/0.062/0.070	0.097/0.069/0.115/0.119/0.121	0.066/0.059/0.077
Resolution range (Å)	41.66–2.80 (2.87–2.80)	41.70–2.35 (2.41–2.35)	41.69–2.90 (2.98–2.90)	74.57–2.30 (2.36–2.30)
Total No. of reflections	192283 (7701)	364259 (18244)	419297 (21915)	888550 (63615)
No. of unique reflections	12297 (883)	20593 (1491)	11149 (816)	22048 (1598)
Completeness (%)	99.8 (99.9)	99.8 (99.8)	99.8 (99.8)	99.9 (100.0)
Multiplicity	15.6 (8.7)	17.7 (12.2)	37.6 (26.9)	40.3 (39.8)
〈*I*/σ(*I*)〉 from merged data	11.11 (1.96)	10.53 (1.86)	12.81 (1.89)	13.29 (2.05)
CC_1/2_	99.5 (74.0)	99.7 (83.7)	99.5 (77.2)	99.8 (83.7)
*R*_meas_	0.240 (1.204)	0.211 (1.145)	0.409 (2.421)	0.352 (2.014)
Overall *B* factor from Wilson plot (Å^2^)	42.4	43.7	41.2	38.2

† As described in the text, three or five isomorphous datasets were merged for each crystal structure.

**Table 4:** Structure refinement Values in parentheses are for the highest resolution shell.

	TNKS2^CAT^+**4**	TNKS2^CAT^+**5**	TNKS2^CAT^+**6**	TNKS2^CAT^+BAD
PDB ID	pdb_00009txt	pdb_00009txu	pdb_00009txv	pdb_00009txw
Ligand ID†	OY6	A1JXX	A1JX7	DQV
Resolution range (Å)	41.66–2.80 (2.87–2.80)	41.70–2.35 (2.41–2.35)	41.69–2.90 (2.98–2.90)	74.57–2.30 (2.36–2.30)
Completeness (%)	99.8 (99.5)	99.8 (99.7)	99.7 (99.8)	99.9 (99.9)
No. of reflections, working set	11070 (788)	18538 (1341)	10035 (736)	19844 (1435)
No. of reflections, test set	1227 (88)	2055 (146)	1114 (77)	2204 (160)
Final *R*_cryst_	0.197 (0.336)	0.212 (0.307)	0.198 (0.321)	0.202 (0.294)
Final *R*_free_	0.241 (0.322)	0.235 (0.328)	0.256 (0.403)	0.241 (0.309)
No. of non-H atoms				
Protein/nucleic acid	3248	3264	3222	3272
Ions	2	2	2	2
Ligands	105	97	95	113
Waters	8	83	0	57
Total	3363	3446	3319	3444
R.m.s. deviations from ideality‡				
Bond lengths (Å)	0.0081	0.0076	0.0085	0.0084
Angles (°)	1.47	1.44	1.51	1.58
Average *B* factors (Å^2^)				
Protein/nucleic acid	48.08	46.89	47.84	42.64
Ions	41.19	42.23	43.62	36.62
Ligands	46.87	66.77	47.44	75.50
Waters	35.00	38.23	n/a	33.24
Ramachandran plot‡				
Favoured regions (%)	95.42	96.68	96.17	98.48
Outliers (%)	0.51	0.51	0.51	0.51
Unmodelled/incomplete residues (%)	8.95	8.72	9.63	8.26

† CCD ID of the inhibitor or BAD.

‡ Values from *MolProbity* (Williams *et al.*, 2018 ▸).

## Data Availability

Atomic coordinates and structure factors have been deposited in the Protein Data Bank (PDB) under accession codespdb_00009txt, pdb_00009txu, pdb_00009txv and pdb_00009txw. Raw diffraction images are available at fairdata.fi (https://doi.org/10.23729/ee61d6dd-5100-4fee-bb21-bf78050d240c).
