# Supplementary material for: Replacement soaking for human tankyrase 2 enables studies on substrate analogues and inhibitors
Source: Acta Crystallogr D Struct Biol. 2026 Jul 29;82(Pt 8):998–1008. doi: 10.1107/S2059798326006868 (PMC13431646; doi:10.1107/S2059798326006868)
Supplement: Supplementary file 1 [file d-82-00998-sup1.pdf]

Acta Cryst D
STRUCTURAL
BIOLOGY

Volume 82 (2026)

Supporting information for article:

Replacement soaking for human tankyrase 2 enables studies on substrate analogues and inhibitors

Johan Pääkkönen, Sven T. Sowa, Chiara Bosetti and Lari Lehtiö

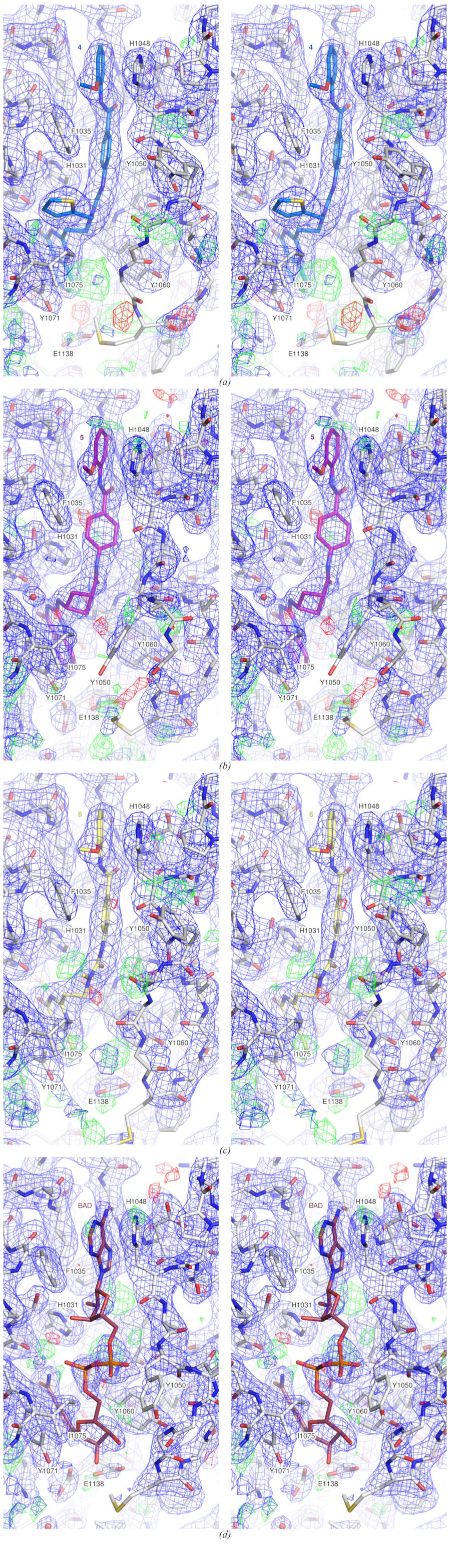

Figure S1 Stereoimages of electron density maps in the donor sites of the TNKS2^{CAT} complex structures with *(a)* **4**, *(b)* **5**, *(c)* **6** and *(d)* BAD as in Fig. 4. The $2mF_o{-}DF_c$ electron density map (blue) is contoured at 1σ, and the $mF_o{-}DF_c$ difference map (green and red) is contoured at $\pm 3\sigma$.
